# Antibacterial and antibiotic-potentiation activities of the methanol extract of some cameroonian spices against Gram-negative multi-drug resistant phenotypes

**DOI:** 10.1186/1756-0500-5-299

**Published:** 2012-06-15

**Authors:** Igor K Voukeng, Victor Kuete, Jean P Dzoyem, Aimé G Fankam, Jaures A K Noumedem, Jules R Kuiate, Jean-Marie Pages

**Affiliations:** 1 Department of Biochemistry, Faculty of science, University of Dschang, P.O. Box 67, Dschang, Cameroon, Africa; 2 Transporteurs Membranaires, Chimiorésistance et Drug Design, UMR-MD1, IFR 88, UFRs de Médecine et de Pharmacie, Marseille, France

**Keywords:** Multi-Drug Resistant bacteria, Spices, Methanol extract, Cameroon

## Abstract

**Background:**

The present work was designed to evaluate the antibacterial properties of the methanol extracts of eleven selected Cameroonian spices on multi-drug resistant bacteria (MDR), and their ability to potentiate the effect of some common antibiotics used in therapy.

**Results:**

The extract of *Cinnamomum zeylanicum* against *Escherichia coli* ATCC 8739 and AG100 strains showed the best activities, with the lowest minimal inhibitory concentration (MIC) of 64 μg/ml. The extract of *Dorstenia psilurus* was the most active when tested in the presence of an efflux pump inhibitor, phenylalanine Arginine*-β-* Naphtylamide (PAβN), a synergistic effect being observed in 56.25 % of the tested bacteria when it was combined with Erythromycin (ERY).

**Conclusion:**

The present work evidently provides information on the role of some Cameroonian spices in the fight against multi-resistant bacteria.

## Background

Infectious diseases are one of the leading causes of morbidity and mortality worldwide, especially in developing countries [1,3]. Following the massive use of antibiotics in human therapy, bacteria have developed several resistance mechanisms including the efflux of antibiotics [3]. Several Cameroonian spices are known to possess medicinal values [4]. In our previous report, we demonstared that several medicinal spices inhibited the growth of MDR bacteria and were also able to improve the activity of commonly used antibiotics [5]. In our continuous search of antimicrobial drugs from medicinal plant, we designed the present work to investigate the antibacterial potential against Gram-negative MDR bacteria of some of the commonly used medicinal spices in Cameroon such as *Aframomum citratum* (Pereira) K. Schum. (Zingiberaceae), *Aframomum melegueta* (Roscoe) K. Schum. (Zingiberaceae), *Scorodophloeus zenkeri* Harms (Caesalpiniaceae), *Tetrapleura tetraptera* (Schum. & Thonn) Taub. (Mimosaceae), *Fagara leprieurii* (Guill and Perr) Engl. (Rutaceae), *Monodora myristica* Dunal (Annonaceae), *Piper guineense* (Schum and Thonn) (Piperaceae), *Dorstenia psilurus* Welwitch (Moraceae), *Imperata cylindricum* Beauv. var. koenigii Durand and Schinz (Gramineae), *Pentadiplandra brazzeana* Baill. (Capparaceae) and *Cinnamomum zeylanicum* (Linn) Cor. (Lauraceae).

## Material and methods

### Plant materials and extraction

The eleven edible spices used in this work were purchased from Dschang local market, West Region of Cameroon in January 2010. The collected spices material were the fruits of *Aframomum citratumAframomum melegueta, Scorodophloeus zenkeri, Tetrapleura tetraptera,* the seeds of *Fagara leprieuriiMonodora myristica* and *Piper guineense*, the roots of *Dorstenia psilurusImperata cylindricum* and *Pentadiplandra brazzeana* and the leaves of *Cinnamomum zeylanicum*. The plants were identified by Mr. Victor Nana of the National herbarium (Yaoundé, Cameroon) where voucher specimens were deposited under a reference number (Table 1). The extracts were obtained by methanol (MeOH) maceration as previously described [5].

**Table 1:** Spices used in the present study and evidence of their activities

**Spice samples (Family)**	**Herbarium Voucher number**^**a**^	**Traditional Treatment**	**Part used**	**Bioactive (or potentially active) compounds**^**b**^**and screened activity**^**c**^**for crude plant extract**
*Aframomum citratum* (Pereira) K. Schum. (Zingiberaceae)	37 736/HNC	Malaria, aphrodisiac, cancer [4,6]	Fruits. leaves. seeds	*Antimicrobial*: Ethylacetate extract of fruits on *Ec. Pa. Sa*[7]
				Cytotoxicity of fruits crude methanol extract [weak activity on leukemia CCRF-CEM and CEM/ADR5000 cells, and pancreatic MiaPaCa-2 cell lines] [4]
*Aframomum melegueta* (Roscoe) K. Schum. (Zingiberaceae)	39 065/HNC	Malaria, dysentery*,* carminative*,* dysmenorrheal*,* fertility, rubella, leprosy, cancer [6,8]	Fruits, leaves	*Antimicrobial* : Aqueous and ethanol extract of leaves on *Fo. An*[9] Methanol extract of fruits *(Q) on Sa. Bs. Ec. Pa. Ca*[8]
				Cytotoxicity of fruits crude methanol extract [weak activity on leukemia CCRF-CEM and pancreatic MiaPaCa-2 cell lines and significant activity on CEM/ADR5000 cells with IC_50_ value of 7.08 μg/ml] [4]
*Cinnamomum zeylanicum* (Linn) Cor. (Lauraceae)	22 309/SRFC	Cancer [4]	Fruits, leaves. bark	*Antimicrobial* : *Cd, Cm, Lt, Fp*[10,11]
				Cytotoxicity of leaves crude methanol extract [weak activity on leukemia CCRF-CEM and CEM/ADR5000 cells, and pancreatic MiaPaCa-2 cell lines] [4]
*Dorstenia psilurus* Welwitch (Moraceae)	44 839/HNC	Snake bite, rheumatism, head and stomach ache, hypertension, cancer [4,12,13].	Leaves, roots	Cytotoxicity of roots crude methanol extract [Significant activity with IC_50_ values of 7.18; 7.79 and 9.17 μg/ml respectively on leukemia CCRF-CEM cells, CEM/5000 cells and pancreatic MiaPaCa-2 cell lines] [4]
*Fagara leprieurii* (Guill and Perr) Engl. (Rutaceae)	37 632/HNC	Gastritis, gingivitis. bilharzias, antidiarrhoeal, cancer, laxative, antimicrobial, ulcer, gonorrhea, kidney ache., sterility [4,14,15]	Bark, leaves. roots	*Antimicrobial* : Ethanol extract of the seeds on *Ca. Cn. Mg. Tm. Tr. Bci. Af. Afl. Sb*[6]Essential oil*: Sa*[15]
				Cytotoxicity of seeds crude methanol extract [weak activity on leukemia CCRF-CEM and pancreatic MiaPaCa-2 cell lines and significant activity on CEM/ADR5000 cells with IC_50_ value of 8.13 μg/ml] [4]
*Imperata cylindricum* Beauv. var. koenigii Durand et Schinz (ramineae)	30 139/SRFC	Diuretic, anti-inflammatory, dysentery, urinary tract infections, cancer [4,16,17]	Leaves, roots	Cytotoxicity of roots crude methanol extract [Significant activity with IC_50_ values of 8.4; 7.18 and 12.11 μg/ml respectively on leukemia CCRF-CEM cells, CEM/5000 cells and pancreatic MiaPaCa-2 cell lines] [4]
*Monodora myristica* Dunal (Annonaceae)	2 949/SRFC	Insecticidal, diuretic, constipation, anti-hemorrhage, headache, wounds, worm infections,cancer [4,15,18,19]	Fruits, leaves. seeds	*Antimicrobial* : *Fm. Afl. Af*[18]; Essential oil*: Af. Bc. Bs. Cgl. Ec. Kp. Sa. Sf*[15]. Cytotoxicity of fruits seeds methanol extract [weak activity on leukemia CCRF-CEM and CEM/ADR5000 cells, and pancreatic MiaPaCa-2 cell lines] [4]
*Pentadiplandra brazzeana* Baill. (Capparaceae)	42 918/HNC	Gastric ulcer, cancer [4,20]	Fruits, leaves	Cytotoxicity of roots crude methanol extract [weak activity on leukemia CCRF-CEM and CEM/ADR5000 cells, and pancreatic MiaPaCa-2 cell lines] [4]
*Piper guineense* (Schum and Thonn) (Piperaceae)	6 018/SRFC	Cough, bronchitis, rheumatism, insecticidal, anemia, carminative, stomach ache, cancer [4,8,21]	Fruits, leaves. bark	Insecticidal : *Cs*[20]*Antimicrobial(Q)*; *Ec. Sa. Bs. Pa. Ca. An*[8,22]
*Scorodophloeus zenkerii* Harms (Caesalpiniaceae)	44 803/HNC	Cancer [4]	Leaves. roots	*Antimicrobial* : Essential oil of stem bark on Ec, Sa, Bs, Cu [23].
				Cytotoxicity of fruits crude methanol extract [weak activity on leukemia CCRF-CEM and CEM/ADR5000 cells, and pancreatic MiaPaCa-2 cell lines] [4]
*Tetrapleura tetraptera* (Schum. & Thonn) Taub. (Mimosaceae)	12 117/SRFC	Pain, arthritis, epilepsy, convulsion, gastric ulcer, cancer [4,20]	Bark, leaves. roots	Cytotoxicity of fruits crude methanol extract [weak activity on leukemia CCRF-CEM and CEM/ADR5000 cells, and pancreatic MiaPaCa-2 cell lines] [4]

^a^(HNC): Cameroon National Herbarium; (SRFC): *Société des reserves forestières du Cameroun*; ^b^(/): Not reported.

^c^[Screened activity: significant (S: CMI < 100 μg/ml). moderate (M : 100 < CMI ≤ 625 μg/ml). Weak (W: CMI > 625 μg/ml) Q: Qualitative activity based on the determination of inhibition zone [15]; Af : *Aspergillus fumigatus*. Afl : *Aspergillus flavus*. An: *Aspergillus niger*. Bc : *Bacillus cereus*. Bci : *Botrytis cinerea*. Bs : *Bacillis subtilis*. Bt: *Botryodiploidia theobromae*. Ca : *Candida albicans*. Cd : *Clostridium difficile*. Cm : *Colletotrichum musae*. Cn : *Cryptococcus neoformans*. Cs: *Callosobruchus subinnotatus*. Cu : *Candida utilis*. Ec : *Escherichia coli*. Fm :*Fusarium moniliforme*. Fo :*Fusarium oxysporum*. Fp : *Fusarium proliferatum*. Lt : *Lasiodiplodia theobromae*. Mg : *Microsporum gypseum*. Pa: *Pseudomonas aeruginosa*. Sa : *Staphylococcus aureus*. Sb: *Scopulariopsis brevicaulis*. Tm: *Trichophyton mentagrophytes*. Tr: *Trichophyton rubrum.*

### Preliminary phytochemical investigations

The major secondary metabolites classes were screened according to the common phytochemical methods described by Harborne [24].

### Chemicals for antimicrobial assays

Tetracycline (TET), cefepime (FEP), streptomycin (STR), ciprofloxacin (CIP), norfloxacin (NOR), chloramphenicol (CHL), cloxacillin (CLX), ampicillin (AMP), erythromycin (ERY), kanamycin (KAN) (Sigma-Aldrich, St Quentin Fallavier, France) were used as reference antibiotic. *p*-Iodonitrotetrazolium chloride (INT) and phenylalanine arginine β-naphthylamide (PAßN) were used as microbial growth indicator and efflux pumps inhibitor (EPI) respectively.

### Bacterial strains and culture media

The studied microorganisms included reference (from the American Type Culture Collection) and clinical (Laboratory collection) strains of *Providencia stuartii, Pseudomonas aeruginosa, Klebsiella pneumoniae, Escherichia coli, Enterobacter aerogenes* and *Enterobacter cloacae* The bacterial strains and their features were previously reported [5]. The preliminary treatment of these organisms as well as the culture media were conducted as previously described [5].

### Bacterial susceptibility determinations

The respective MICs of samples on the studied bacteria were determined using rapid INT colorimetric assay [25,26] with some modifications as previously reported [5]. The inoculum concentration used was 1.5 x10^6^ CFU/ml and the samples were incubated at 37 °C for 18 h [5]. The final concentration of DMSO was lower than 2.5 % and this concentration also served as negative control [5]. Chloramphenicol was used as reference antibiotic. The MICs of samples were detected after 18 h incubation at 37 °C, following addition (40 μl) of 0.2 mg/ml INT and incubation at 37 °C for 30 minutes [5]. MIC was defined as the lowest sample concentration that prevented the color change of the medium and exhibited complete inhibition of microbial growth [27].

Samples were tested alone and then, in the presence of PAßN at 20 mg/L final concentration as previously reported [5]. Four of the best extracts, those from *A. citratum, C. zeylanicum, D. psilurus* and *T. tetraptera* were also tested in association [5] at the concentrations selected following a preliminary assay on *P. aeruginosa* PA124 (See Additional file 1: Table S1). All assays were performed in triplicate and repeated thrice. Fractional inhibitory concentration (FIC) [5] were calculated and the interpretations were made as follows: synergistic (*<*0.5), indifferent (0.5 to 4), or antagonistic (*>*4) [28] (The FIC values available in Additional file 1: Table S2 and S3).

## Results

### Phytochemical composition of the spice extracts

The results of qualitative analysis showed that each plant contains various phytochemicals compounds such as alkaloids, anthocyanins, anthraquinones, flavonoids, phenols, saponins, steroids, tannins and triterpenes as shown in Table 2.

**Table 2:** Extraction yields, aspects and phytochemical composition of the plant extracts

Spice samples	Extraction yield (%)*	Physical aspect	Phytochemical composition								
			Alkaloids	Anthocyanins	Anthraquinons	Flavonoids	Phenols	Saponins	Sterols	Tannins	Triterpenes
*Aframomum citratum*	2.6	Oily, dark green	+	-	-	+	+	-	-	+	+
*Aframomum melegueta*	7.3	Brown powder	+	-	-	-	-	+	-	-	+
*Cinnamomum zeylanicum*	8.4	Oily, dark green	+	-	-	+	+	-	+	+	-
*Dorstenia psilurus*	10.3	Oily, brown	+	+	+	+	+	+	-	+	+
*Fagara leuprieurii*	26.2	Creamy, brown	+	-	+	+	+	-	-	+	+
*Imperata cylindricum*	8.2	Creamy, brown	+	+	+	+	+	-	-	-	+
*Monodora myristica*	23.5	Oily, brown	+	-	+	+	+	-	-	-	+
*Pentadiplandra brazzeana*	4.6	Creamy, brown	+	-	-	+	+	-	-	-	-
*Piper guineense*	17.5	Creamy, brown	+	-	+	+	+	-	-	-	-
*Scorodophloeus zenkeri*	9.2	Creamy, dark green	+	-	-	+	+	-	-	+	-
*Tetrapleura tetraptera*	29.4	brown	+	-	+	+	+	+	-	+	+

(+): Present; (−): Absent; *The yield was calculated as the ratio of the obtained methanol extract according to the initial mass of the spice powder.

### Antibacterial activity of the spice extracts

The results summarized in Table 3 summarize the MIC of the extract tested alone or in combination with PAβN on the tested microorganisms. Its shows that all the studied extracts were active on at least one microbial strain. *A. citratum* showed the best activity, it inhibitory effect being recorded on 85% (24/28) of the tested bacteria. Other samples were less active, their inhibitory potencies being observed on 75% of tested bacteria (21/28) for *I*. *cylindricum* and *C*. *zeylanicum*, 67.9 % (19/28) for *A. melegueta*, *D. psilurus*, *F. leprieuri* and *T. tetraptera*; 64.3% (18/28) for *M. myristica* and *S. zenkeri*; 50 % (14/28) for *P. guineense* and 42.9 % (12/28) for *P. brazzeana*.

**Table 3:** Minimal inhibitory concentration (MIC) of the studied plants extracts and chloramphenicol on the studied bacterial species

**Bacterial strains**	**Tested samples and MIC in μg/ml in the absence and presence of PAßN (in parenthesis)**											
	***Aframomum citratum***	***Aframomum melegueta***	***Imperata cylindricum***	***Cinnamomum zeylanicum***	***Dorstenia psilurus***	***Fagara leprieuri***	***Monodora myristica***	***Pentadiplandra brazzeana***	***Piper guineense***	***Scorodophloeus zenkeri***	***Tetrapleura tetraptera***	**CHL**
***E. coli***												
ATCC8739	512	512	512	**64**	-	512	1024	1024	1024	1024	1024	1
ATCC10536	1024	512	1024	512	128	256	1024	512	1024	512	1024 (1024)	32 (<2)
AG100	1024 (1024)	1024 (1024)	1024 (256)	- **(64)**	- (1024)	1024 (1024)	512	1024 (1024)	1024 (512)	1024 (1024)	1024 (1024)	4 (<2)
AG100A	512 (128)	1024 (1024)	1024 (128)	512 (128)	512 (128)	512 (512)	1024 (1024)	- (−)	1024 (1024)	1024 (1024)	1024 (1024)	<2 (<2)
AG100A_TET_	512 (512)	1024 (1024)	1024 (1024)	512 (512)	512 (128)	1024 (1024)	-	-	1024	512	1024	32 (<2)
AG102	1024	-	1024	1024	512	1024	-	-	-	-	-	16 (<2)
MC4100	512 (512)	512 (256)	1024 (1024)	1024 (1024)	512 (256)	512	- (−)	1024 (1024)	1024 (1024)	1024 (1024)	512 (512)	4 (<2)
W3110	512 (256)	512 (512)	512 (512)	512 (512)	512 (256)	256	512	1024 (1024)	1024 (128)	512	512 (512)	1 (<2)
***E. aerogenes***												
ATCC13048	1024	-	1024	1024	1024	1024	1024	-	-	-	-	8 (<2)
CM64	1024 (1024)	1024 (1024)	512 (128)	1024 (512)	512 (256)	1024 (1024)	1024 (1024)	1024 (1024)	1024 (1024)	1024 (1024)	512 (512)	32
EA27	512 (512)	1024 (1024)	512 (512)	512 (512)	- (−)	1024 (1024)	1024 (1024)	1024 (1024)	-(−)	1024 (1024)	1024 (512)	64 (32)
EA289	-	1024	-	1024	-	-	1024	1024	-	-	1024	256
EA298	1024	512	-	-	1024	-	256	256	512	256	1024	256
EA3	-	-	-	-	-	1024	-	-	-	-	-	256
***E. cloacae***												
BM47	512 (512)	1024 (1024)	1024 (1024)	1024 (512)	1024 (128)	1024 (1024)	1024 (1024)	1024	1024	1024	1024	- (8)
BM67	512 (512)	1024 (1024)	1024 (1024)	1024 (1024)	1024 (128)	- (−)	- (−)	- (−)	- (−)	- (−)	- (−)	- (32)
ECCI69	512 (512)	1024 (1024)	1024 (1024)	1024 (1024)	-(−)	-(−)	1024 (1024)	- (−)	1024 (1024)	1024 (1024)	1024 (512)	- (32)
***K. pneumoniae***												
ATCC12296	1024	1024	1024	1024	1024	1024	512	-	-	-	1024	4
K2	1024	-	1024	1024	1024	-	1024	-	-	-	-	-
K24	1024	1024	1024	1024	1024	1024	512	-	-	1024	1024	32 (<2)
KP55	512	1024	256	512	512	1024	1024	-	-	-	1024	32(<2)
KP63	512 (512)	1024 (1024)	1024 (1024)	512 (512)	512 (128)	1024 (512)	1024 (1024)	512	1024 (1024)	1024 (512)	1024 (1024)	64(<2)
***P. stuartuii***												
ATCC29916	1024 (1024)	- (−)	-(−)	-(−)	1024 (1024)	-(−)	-(−)	-(−)	1024 (1024)	1024 (1024)	1024 (1024)	8
NEA16	1024 (512)	- (−)	1024 (1024)	512 (512)	512 (256)	1024	1024	-	-	1024	-	64(<2)
PS2636	1024	-	-	-	-	-	-	-	-	-	-	-
PS299645	512	512	1024	1024	1024	1024	-	1024	1024	512	1024	128
***P. aeruginosa***												
PA01	-	-	-	-	-	-	-	-	-	-	-	-
PA124	-	-	-	-	-	-	-	-	-	1024	-	32(<2)

(−): MIC not detected at up to 1024 μg/ml for the les extracts and 256 μg/ml for chloramphenicol. **()** : values in parenthesis are MIC of substance in the presence of PAßN at 20 μg/ml. The MIC of PAßN was 64 μg/ml on *E. coli*. AG100A. 512 μg/ml on ATCC11296. BM67. EA27. EA289; 1024 μg/ml on AG100A_TET._ ATCC13048. CM64; and > 1024 μg/ml on other bacteria. CHL**:** chloramphénicol; (in bold): significant MIC value.

### Role of efflux pumps in susceptibility of gram negative bacteria to the tested spice extracts

Potentiating effect of EPI was not observed on tested bacteria when associated with *M. myristica*, *P. brazzeana*, *T. tetraptera* and *S. zenkeri*. PAβN weakly increased the activity of *A. citratum*, *A. melegueta*, *F. leprieuri*, *I*. *cylindricum*, *C*. *zeylanicum* and *P. guineense*. The activity of *D. psilurus* in the presence of EPI significantly increased on most of the tested bacteria (except against *P. stuartii* ATCC29916, *E. cloacae* ECCI69 and *E. aerogenes* EA27) (see Table 3).

### Effects of the association of some spice extracts with antibiotics

*A. citratum*, *C*. *zeylanicum*, *D. psilurus* and *T. tetraptera* (Tables 4, 5, 6 and 7) were associated to antibiotics in view of evaluating the possible synergistic effect of these associations. A preliminary study using *P. aeruginosa* PA124 was carried out with ten antibiotics (CLX, AMP, ERY, KAN, CHL, TET, FEP, STR, CIP and NOR) to select the appropriate sub-inhibitory concentrations to be used. MIC/2.5 and MIC/5 were then selected as the sub-inhibitory concentrations (see Additional file 1: Table S1). All of these four extracts were then tested in association with antibiotics previously listed on strains of *E. coli* AG100A_TET_ and AG102, *E. aerogenes* CM64, *K. pneumonia* KP63 and *P. aeruginosa* PA124. No antagonistic effect (FIC > 4) was observed between extracts and antibiotics meanwhile indifference was observe between *T. tetraptera* and antibiotics in most of the case (see Tables 5, 6, and 7, Additional file 1: S2, S3, S4 and S5). Significant increase of the activity was observed with the association of the extracts of *A. citratum* and *D. psilurus* on *E. aerogenes* CM64 and *K. pneumoniae* KP63, and with *C. zeylanicum* against *K. pneumoniae* KP63. A significant decrease (synergy effect) of MIC values was also observed when ERY was associated with various extracts, and when extracts of *A. citratum* and *C. zeylanicum* were each combined with aminoglycosides (KAN, STR), the best activity being noted against *E. aerogenes* CM64.

**Table 4:** **Minimal inhibitory concentration (MIC) in μg/ml of antibiotics in the absence and presence sub-inhibitory concentrations of*Aframomum citratum*extract against some MDR bacteria**

**Bacterial strains**	**Antibiotics and MIC in absence and presence of*Aframomum citratum*extract**														
	**Ampicillin**			**Cefepime**			**Chloramphenicol**			**Ciprofloxacin**			**Cloxacillin**		
	**Alone**	**MIC/2.5**	**MIC/5**	**Alone**	**MIC/2.5**	**MIC/5**	**Alone**	**MIC/2.5**	**MIC/5**	**Alone**	**MIC/2.5**	**MIC/5**	**Alone**	**MIC/2.5**	**MIC/5**
AG100Atet	-	-	-	-	-	-	256	32 (8) ^S^	64 (4) ^S^	256	128 (2) ^S^	256 (1) ^I^	-	-	-
AG102	-	-	-	128	128 (1) ^I^	128 (1) ^I^	16	8 (2) ^S^	8 (2) ^S^	<2	<2	<2	-	-	-
CM64	-	256 (1)^I^	-	-	64 (>4) ^S^	-	nt	nt	nt	nt	nt	nt	-	-	-
KP63	-	32 (>8)^S^	-	256	32 (8) ^S^	256 (1) ^I^	-	64 (>4) ^S^	256 (>1)^S^	64	64 (1) ^I^	64 (1) ^I^	-	256 (>1)^S^	-
PA124	128	16 (8) ^S^	64 (2) ^S^	128	128 (1) ^I^	256 (0.5) ^I^	32	16 (2) ^S^	16 (2) ^S^	16	4 (4) ^S^	16 (1) ^I^	-	-	-
**Bacterial strains**	**Erythromycin**			**Kanamycin**			**Norfloxacin**			**Streptomycin**			**Tetracyclin**		
	**Alone**	**MIC/2.5**	**MIC/5**	**Alone**	**MIC/2.5**	**MIC/5**	**Alone**	**MIC/2.5**	**MIC/5**	**Alone**	**MIC/2.5**	**MIC/5**	**Alone**	**MIC/2.5**	**MIC/5**
AG100Atet	64	16 (4) ^S^	32 (2) ^S^	-	32 (>8)^S^	256	128	16 (8) ^S^	128 (1) ^I^	<2	<2	<2	2	<2 (>1)^S^	2 (1) ^I^
AG102	32	16 (2) ^S^	16 (2) ^S^	<2	<2	<2	<2	<2	<2	-	128 (>2)^S^	256 (>1)^S^	<2	<2	<2
CM64	-	128 (>2)^S^	256 (>1)^S^	4	<2	<2	4	<2 (>2)^S^	4 (1) ^I^	32	4 (8) ^S^	8 (4) ^S^	nt	nt	nt
KP63	16	<1 (>16)^S^	4 (4) ^S^	32	16 (2) ^S^	32 (1) ^I^	-	128 (>2)^S^	256 (>1)^S^	<4	<4	<4	<2	<2	<2
PA124	128	64 (2) ^S^	64 (2) ^S^	128	16 (8) ^S^	64 (2) ^S^	64	8 (8) ^S^	32 (2) ^S^	nt	nt	nt	8	2 (4) ^S^	2 (4) ^S^

MIC/2.5: concentration of plant extract added equal to 204.8 μg/mL for AG100A_TET_. KP63; and to 409.6 μg/mL for PA124. CM64. AG102.

MIC/5: concentration of plant extract added equal to 102. 4 μg/mL for AG100A_TET_. KP63; and to 204.8 μg/mL for PA124. CM64. AG102.

(): Folds decreasing of MIC. S: synergy. I: indifference. nt: not tested; (−): MIC > 256.

**Table 5:** **Minimal inhibitory concentration (MIC) of antibiotics in absence and presence of*Cinnamomum zeylanicum*extract (μg/mL)**

**Bacterial strains**	**Antibiotics and MIC in absence and presence of*Cinnamomum zeylanicum*extract**														
	**Ampicillin**			**Cefepime**			**Chloramphenicol**			**Ciprofloxacin**			**Cloxacillin**		
	**Alone**	**MIC/2.5**	**MIC/5**	**Alone**	**MIC/2**	**MIC/5**	**Alone**	**MIC/2.5**	**MIC/5**	**Alone**	**MIC/2.5**	**MIC/5**	**Alone**	**MIC/2.5**	**MIC/5**
AG100Atet	-	-	-	-	-	-	256	16 (16) ^S^	32 (8) ^S^	256	64 (4) ^S^	128 (2) ^S^	-	-	-
AG102	-	-	-	128	256 (0.5) ^I^	256 (1) ^I^	16	8 (2) ^S^	16 (1) ^I^	<2	<2	<2	-	256 (>1)^S^	-
CM64	-	256 (>1) ^S^	-	-	256 (>1) ^S^	-	nt	nt	nt	nt	nt	nt	-	-	-
KP63	-	32 (>8) ^S^	-	256	32 (8) ^S^	256 (1) ^I^	-	32 (>8) ^S^	256 (>1) ^S^	64	128 (0.5) ^I^	128(0.5)^I^	-	64 (>4)^S^	256 (>1)^S^
PA124	128	16 (8) ^S^	64 (2) ^S^	128	128 (1) ^I^	128 (1) ^I^	32	2 (16) ^S^	8 (4) ^S^	16	8 (2) ^S^	16 (1) ^I^	-	-	-
**Bacterial strains**	**Erythromycin**			**Kanamycin**			**Norfloxacin**			**Streptomycin**			**Tetracycline**		
	**Alone**	**MIC/2.5**	**MIC/5**	**Alone**	**MIC/2.5**	**MIC/5**	**Alone**	**MIC/2.5**	**MIC/5**	**Alone**	**MIC/2.5**	**MIC/5**	**Alone**	**MIC/2.5**	**MIC/5**
AG100Atet	64	16 (4) ^S^	32 (2) ^S^	-	16 (>16)^S^	128 (>2)^S^	128	128 (1) ^I^	128 (1) ^I^	<2	<2	<2	2	2 (1) ^I^	2 (1) ^I^
AG102	32	16 (2) ^S^	16 (2) ^S^	<2	<2	<2	<2	<2	<2	-	256 (>1) ^S^	256 (>1)^S^	<2	<2	<2
CM64	-	128 (>2)^S^	256 (>1)^S^	4	<2 (>2) ^S^	<2 (>2) ^S^	4	<2 (>2) ^S^	4 (1) ^I^	32	4 (8) ^S^	8 (4) ^S^	nt	nt	nt
KP63	16	1 (16) ^S^	4 (4) ^S^	32	32 (1) ^I^	32 (1) ^I^	-	128 (>2)^S^	256 (>1)^S^	<4	<4	<4	<2	<2	<2
PA124	128	16 (8) ^S^	32 (4) ^S^	128	8 (16) ^S^	32 (4) ^S^	64	32 (2) ^S^	64 (1) ^I^	nt	nt	nt	8	2 (4) ^S^	2 (4) ^S^

MIC/2.5: concentration of plant extract added equal to 204.8 μg/mL for AG100A_TET_. KP63; and to 409.6 μg/ml for PA124. CM64. AG102.

MIC/5: concentration of plant extract added equal to 102. 4 μg/mL for AG100A_TET_. KP63; and to 204.8 μg/ml for PA124. CM64. AG102.

(): Folds decreasing of MIC. S: synergy. I: indifference. nt: not tested; (−): MIC > 256 μg/ml.

**Table 6:** **Minimal inhibitory concentration (MIC) of antibiotics in absence and presence extracts*Dorstenia psilurus*(μg/ml)**

**Bacterial strains**	**Antibiotics and MIC in absence and presence of*Dorstenia psilurus*extract**														
	**Ampicillin**			**Cefepime**			**Chloramphenicol**			**Ciprofloxacin**			**Cloxacillin**		
	**Alone**	**MIC/2.5**	**MIC/5**	**Alone**	**MIC/2**	**MIC/5**	**Alone**	**MIC/2.5**	**MIC/5**	**Alone**	**MIC/2.5**	**MIC/5**	**Alone**	**MIC/2.5**	**MIC/5**
AG100Atet	-	-	-	-	-	-	256	128 (2) ^S^	256 (1) ^I^	256	64 (4) ^S^	128 (2) ^S^	-	-	-
AG102	-	-	-	128	256 (0.5) ^I^	256 (0.5) ^I^	16	4 (4) ^S^	4 (4) ^S^	<2	<2	<2	-	-	-
CM64	-	256 (>1)^S^	-	-	64 (>4) ^S^	64 (>4) ^S^	nt	nt	nt	nt	nt	nt	-	256 (>1) ^S^	-
KP63	-	32 (>8) ^S^	-	256	64 (4) ^S^	128 (2) ^S^	-	64 (>4) ^S^	256 (>1) ^S^	64	64 (1) ^I^	64 (1) ^I^	-	64 (>4) ^S^	256 (>1) ^S^
PA124	128	64 (2) ^S^	64 (2) ^S^	128	128 (1) ^I^	128 (1) ^I^	32	16 (2) ^S^	32 (1) ^I^	16	16 (1) ^I^	16 (1) ^I^	-	-	-
**Bacterial strains**	**Erythromycin**			**Kanamycin**			**Norfloxacin**			**Streptomycin**			**Tetracycline**		
	**Alone**	**MIC/2.5**	**MIC/5**	**Alone**	**MIC/2.5**	**MIC/5**	**Alone**	**MIC/2.5**	**MIC/5**	**Alone**	**MIC/2.5**	**MIC/5**	**Alone**	**MIC/2.5**	**MIC/5**
AG100Atet	64	32 (2) ^S^	32 (2) ^S^	-	128 (>2)^S^	256 (>1) ^S^	128	128 (1) ^I^	256 (0.5) ^I^	<2	<2	<2	2	2 (1) ^I^	2 (1) ^I^
AG102	32	64 (0.5) ^I^	64 (0.5) ^I^	<2	<2	<2	<2	<2	<2	-	256 (>1)^S^	256 (>1)^S^	<2	<2	<2
CM64	-	64 (>4) ^S^	256 (>1)^S^	4	4 (1) ^I^	8 (0.5) ^I^	4	<2 (>2)^S^	<2 (>2)^S^	32	8 (4) ^S^	32 (1) ^I^	nt	nt	nt
KP63	16	1 (16) ^S^	8 (2) ^S^	32	32 (1) ^I^	64 (0.5) ^I^	-	-	-	<4	<4	<4	<2	<2	<2
PA124	128	64 (2) ^S^	128 (1) ^I^	128	4 (32) ^S^	16 (8) ^S^	64	32 (2) ^S^	64 (1) ^I^	nt	nt	nt	8	2 (4) ^S^	8 (1) ^I^

MIC/2.5: concentration of plant extract added equal to 204.8 μg/mL for AG100A_TET_. CM64. KP63. AG102 and to 409.6 μg/ml for PA124.

MIC/5: concentration of plant extract added equal to 102. 4 μg/mL for AG100A_TET_ CM64. KP63. AG102; and to 204.8 μg/ml for PA124.

(): Folds decreasing of MIC. S: synergy. I: indifference. nt: not tested; (−): MIC > 256 μg/ml.

**Table 7:** **Minimal inhibitory concentration (MIC) of antibiotics in absence and presence extracts*Tetrapleura tetraptera*(μg/ml)**

**Bacterial strains**	**Antibiotics and MIC in absence and presence*Tetrapleura tetraptera*extract**														
	**Ampicillin**			**Cefepime**			**Chloramphenicol**			**Ciprofloxacin**			**Cloxacillin**		
	**Alone **	**MIC/2.5**	**MIC/5**	**Alone **	**MIC/2**	**MIC/5**	**Alone **	**MIC/2.5**	**MIC/5**	**Alone **	**MIC/2.5**	**MIC/5**	**Alone **	**MIC/2.5**	**MIC/5**
AG100Atet	-	-	-	-	-	-	256	256 (1) ^I^	-	256	128 (2) ^S^	128 (2)^S^	-	-	-
AG102	-	-	-	128	256 (0.5)^I^	256 (0.5) ^I^	16	8 (2) ^S^	8 (2) ^S^	<2	<2	<2	-	-	-
CM64	-	-	-	-	-	-	nt	nt	nt	nt	nt	nt	-	-	-
KP63	-	-	-	256	256 (1) ^I^	-	-	256 (>1) ^S^	256 (>1) ^S^	64	64 (1) ^I^	64 (1) ^I^	-	128 (>2) ^S^	256 (>1) ^S^
PA124	128	64 (2)^S^	128 (1)^I^	128	128 (1)^I^	128 (1) ^I^	32	4 (8) ^S^	8 (4) ^S^	16	16 (1) ^I^	16 (1) ^I^	-	-	-
**Bacterial strains**	**Erythromycin**			**Kanamycin**			**Norfloxacin**			**Streptomycin**			**Tetracycline**		
	**Alone**	**MIC/2.5**	**MIC/5**	**Alone**	**MIC/2.5**	**MIC/5**	**Alone**	**MIC/2.5**	**MIC/5**	**Alone**	**MIC/2.5**	**MIC/5**	**Alone**	**MIC/2.5**	**MIC/5**
AG100Atet	64	64 (1) ^I^	64 (1) ^I^	-	256 (>1) ^S^	256 (>1) ^S^	128	128 (1) ^I^	256 (0.5) ^I^	<2	<2	<2	2	2 (1) ^I^	2 (1) ^I^
AG102	32	64 (0.5) ^I^	64 (0.5) ^I^	<2	<2	<2	<2	<2	<2	-	256 (>1) ^S^	256 (>1) ^S^	<2	<2	<2
CM64	-	256 (>1) ^S^	-	4	4 (1) ^I^	8 (0.5) ^I^	4	4 (1) ^I^	8 (0.5) ^I^	32	16 (2) ^S^	32 (1) ^I^	nt	nt	nt
KP63	16	<1 (>16) ^S^	8 (2) ^S^	32	32 (1) ^I^	64 (0.5) ^I^	-	256 (>1) ^S^	-	<4	<4	<4	<2	<2	<2
PA124	128	64 (2) ^S^	64 (2) ^S^	128	64 (2) ^S^	64 (2) ^S^	64	32 (2) ^S^	64 (1) ^I^	nt	nt	nt	8	2 (4) ^S^	2 (4) ^S^

MIC/2.5: concentration of plant extract added equal to 204.8 μg/mL for CM64 and to 409.6 μg/ml for AG100A_TET_. PA124. KP63. AG102.

MIC/5: concentration of plant extract added equal to 102. 4 μg/mL for CM64; and to 204.8 μg/ml for AG100A_TET_.PA124. KP63. AG102.

(): Folds decreasing of MIC. S: synergy. I: indifference. nt: not tested; (−): MIC > 256 μg/ml.

## Discussion

### Phytochemical composition of the spice extracts

The phytochemical studies revealed the presence of secondary metabolite such as alkaloids, anthocyanins, anthraquinones, flavonoids, phenols, saponins, sterols, tannins and triterpenes; several molecules belonging to these classes of secondary metabolites were found active on pathogenic microorganisms [29].

### Antibacterial activity of the spice extract

Although this is the first time that plants used in this work are studied for their activities vis-à-vis multi-resistant bacteria, plants belonging to some of the genus studied herein, like the *Aframomum* genus are well documented for their antimicrobial activity [6]. Some antibacterial compounds, such as acridone and chelerythrine have previously been isolated from the fruits of *F. leprieurii*[14,30]. The antimicrobial activity of *P. brazzeana* and *S. zenkeri* is mainly due to some sulfur compounds. In fact, sulfur compounds with antimicrobial properties have previously been isolated from the two plants [7,31]. Several alkaloids of the genus *Piper* proved to be responsible for the activity of *P. guineense*[32]. The detection of this class of secondary metabolites in the extract studied herein can explain the observed activities. According to Krishnaiah et al. [16], the antimicrobial activity of *I. cylindricum* can be due to the presence of tannins in this plant. However, tannins were not detected in the extract of *I. cylindricum* as found in the present work (Table 2), suggesting that other classes of secondary metabolites might be responsible for the antibacterial activity of this plant.

### Role of efflux pumps in susceptibility of gram negative bacteria to the tested spice extracts

The significant increase of the activity of the extract of *D. psilurus* in the presence of EPI, indicates that bioactive constituents of this plant extract are substrate of efflux pumps. Efflux through AcrAB-TolC pumps was reported as essential mode of resistance of several Gram-negative MDR bacteria to a number of flavonoids isolated from plants of the genus *Dorstenia*, such as isobavachalcone, kanzonol C, stipulin, etc. [4,15,33-35]. This suggests that possible combination of the extract of *D. psilurus* with EPI can be envisaged to overcome MDR bacteria.

### Effects of the association of extracts with antibiotics

The results obtained by combining the antibiotic with the extracts of *A. citratum*, *C. zeylanicum*, *D. psilurus* and *T. tetraptera* indicate that these extracts contain chemical compounds that can modulate the activity of antibiotics against bacteria expressing MDR phenotypes. The methanol extracts of *A. citratum*, *C. zeylanicum* and *D. psilurus* showed a synergistic effect with antibiotics inhibiting bacterial cell wall synthesis (AMP and CEF) on *K. pneumoniae* KP63. The intrinsic mode of action of the active extracts is to be investigated.

## Conclusion

The present work evidently provides information in the role of some Cameroonian spices in the fight against multi-resistant bacteria. The study also highlights the potential of *D. psilurus* as a strong antibacterial agent when the extract is combined with efflux pump inhibitor and several antibiotics.

## Competing interest

The authors declare that they have no competing interest.

## Authors’ contributions

IKV carried out the study; VK designed the experiments and wrote the manuscript; VK, GAF, JAKN, JPD, JRK and JMP supervised the work; VK and JMP provided the bacterial strains; All authors read and approved the final manuscript.

## Supplementary Material

Additional file 1 Table S1.Activities of antibiotics in combination with the sub-inhibitory concentrations of some plants extracts on *Pseudomonas aeruginosa* PA124. **S2.** Fractional inhibitory Concentrations of the association between antibiotics and extracts of *Aframomum citratum* at MIC/2.5 and MIC/5 (μg/ml) against MDR bacteria. **S3.** Fractional inhibitory Concentrations of the association between antibiotics and extracts of *Cinnamomum zeylanicum* at MIC/2.5 and MIC/5 (μg/ml) against MDR bacteria. **S4.** Fractional inhibitory Concentrations of the association between antibiotics and extracts of *Dorstenia psilurus* at MIC/2.5 and MIC/5 (μg/ml) against MDR bacteria. **S5.** Fractional inhibitory Concentrations of the association between antibiotics and extracts of *Tetrapleura tetraptera* at MIC/2.5 and MIC/5 (μg/ml) against MDR bacteria. Click here for file
