# Supplementary material for: Antibacterial and antibiotic-potentiation activities of the methanol extract of some cameroonian spices against Gram-negative multi-drug resistant phenotypes
Source: BMC Res Notes. 2012 Jun 15;5:299. doi: 10.1186/1756-0500-5-299 (PMC3438083; doi:10.1186/1756-0500-5-299)
Supplement: Additional file 1 — Table S1.Activities of antibiotics in combination with the sub-inhibitory concentrations of some plants extracts on Pseudomonas aeruginosa PA124. S2. Fractional inhibitory Concentrations of the association between antibiotics and extracts of Aframomum citratum at MIC/2.5 and MIC/5 (μg/ml) against MDR bacteria. S3. Fractional inhibitory Concentrations of the association between antibiotics and extracts of Cinnamomum zeylanicum at MIC/2.5 and MIC/5 (μg/ml) against MDR bacteria. S4. Fractional inhibitory Concentrations of the association between antibiotics and extracts of Dorstenia psilurus at MIC/2.5 and MIC/5 (μg/ml) against MDR bacteria. S5. Fractional inhibitory Concentrations of the association between antibiotics and extracts of Tetrapleura tetraptera at MIC/2.5 and MIC/5 (μg/ml) against MDR bacteria. [file 1756-0500-5-299-S1.doc]

**Supplemental materials (S)**

S1. Activities of antibiotics in combination with the sub-inhibitory concentrations of some plants extracts on *Pseudomonas aeruginosa* PA124.

| **Extract and concentrations** | | **Antibiotics and MIC (µg/ml)** | | | | | | | | | | | | | | | | | |
| --- | --- | --- | --- | --- | --- | --- | --- | --- | --- | --- | --- | --- | --- | --- | --- | --- | --- | --- | --- |
|  |  | **AMP** | | **FEP** | | **CHL** | | **CIP** | | **CLX** | | **ERY** | | **KAN** | | **NOR** | | **TET** | |
|  | Alone | 128 | | 128 | | 32 | | 16 | | >512 | | 128 | | 128 | | 64 | | 8 | |
| *Aframomum* *citratum* | MIC/2.5 | | 16 (0.125) ^S^ | | 128 (1) ^I^ | | 16 (0.5) ^S^ | | 4 (0.25) ^S^ | | >512 | | 64 (0.5) ^S^ | | 16 (0.125) ^S^ | | 8 (0.125) ^S^ | | 2 (0.25) ^S^ |
|  | MIC/5 | | 64 (0.5) ^S^ | | 256 (2) ^I^ | | 16 (0.5) ^S^ | | 16 (1) ^I^ | | >512 | | 64 (0.5) ^S^ | | 64 (0.5) ^S^ | | 32 (0.5) ^S^ | | 2 (0.25) ^S^ |
|  | MIC/10 | | 64 (0.5) ^S^ | | 256 (2) ^I^ | | 32 (1) ^I^ | | 16 (1) ^I^ | | >512 | | 64 (0.5) ^S^ | | 64 (0.5) ^S^ | | 64 (1) ^I^ | | 4 (0.5) ^S^ |
|  | MIC/20 | | 128 (1) ^I^ | | 256 (2) ^I^ | | 32 (1) ^I^ | | 16 (1) ^I^ | | >512 | | 128 (1) ^I^ | | 128 (1) ^I^ | | 64 (1) ^I^ | | 4 (0.5) ^S^ |
| *Cinnamomum* *zeylanicum* | MIC/2.5 | | 16 (0.125) ^S^ | | 128 (1) ^I^ | | 2 (0.063) ^S^ | | 8 (0.5) ^S^ | | 512 | | 16 (0.125) ^S^ | | 8 (0.063) ^S^ | | 32 (0.5) ^S^ | | 2 (0.25) ^S^ |
|  | MIC/5 | | 64 (0.5) ^S^ | | 128 (1) ^I^ | | 8 (0.125) ^S^ | | 16 (1) ^I^ | | >512 | | 32 (0.25) ^S^ | | 32 (0.25) ^S^ | | 64 (1) ^I^ | | 2 (0.25) ^S^ |
|  | MIC/10 | | 64 (0.5) ^S^ | | 128 (1) ^I^ | | 16 (0.5) ^S^ | | 16 (1) ^I^ | | >512 | | 64 (0.5) ^S^ | | 128 (1) ^I^ | | 64 (1) ^I^ | | 4 (0.5) ^S^ |
|  | MIC/20 | | 64 (0.5) ^S^ | | 128 (1) ^I^ | | 32 (1) ^I^ | | 16 (1) ^I^ | | >512 | | 64 (0.5) ^S^ | | 128 (1) ^I^ | | 64 (1) ^I^ | | 4 (0.5) ^S^ |
| *Dorstenia* *psilurus* | MIC/2.5 | | 64 (0.5) ^S^ | | 128 (1) ^I^ | | 16 (0.5) ^S^ | | 16 (1) ^I^ | | >512 | | 64 (0.5) ^S^ | | 4 (0.031) ^S^ | | 32 (0.5) ^S^ | | 2 (0.25) ^S^ |
|  | MIC/5 | | 64 (0.5) ^S^ | | 128 (1) ^I^ | | 32 (1) ^I^ | | 16 (1) ^I^ | | >512 | | 128 (1) ^I^ | | 16 (0.063) ^S^ | | 64 (1) ^I^ | | 8 (1) ^I^ |
|  | MIC/10 | | 128 (1) ^I^ | | 128 (1) ^I^ | | 32 (1) ^I^ | | 16 (1) ^I^ | | >512 | | 128 (1) ^I^ | | 64 (0.5) ^S^ | | 64 (1) ^I^ | | 8 (1) ^I^ |
|  | MIC/20 | | 128 (1) ^I^ | | 128 (1) ^I^ | | 32 (1) ^I^ | | 16 (1) ^I^ | | >512 | | 128 (1) ^I^ | | 64 (0.5) ^S^ | | 64 (1) ^I^ | | 8 (1) ^I^ |
| *Tetrapleura* *tetraptera* | MIC/2.5 | | 64 (0.5) ^S^ | | 128 (1) ^I^ | | 4 (0.125) ^S^ | | 16 (1) ^I^ | | >512 | | 64 (0.5) ^S^ | | 64 (0.5) ^S^ | | 32 (0.5) ^S^ | | 2 (0.25) ^S^ |
|  | MIC/5 | | 128 (1) ^I^ | | 128 (1) ^I^ | | 8 (0.25) ^S^ | | 16 (1) ^I^ | | >512 | | 64 (0.5) ^S^ | | 64 (0.5) ^S^ | | 64 (1) ^I^ | | 2 (0.25) ^S^ |
|  | MIC/10 | | 128 (1) ^I^ | | 128 (1) ^I^ | | 32 (1) ^I^ | | 16 (1) ^I^ | | >512 | | 64 (0.5) ^S^ | | 64 (0.5) ^S^ | | 64 (1) ^I^ | | 4 (0.5) ^S^ |
|  | MIC/20 | | 128 (1) ^I^ | | 128 (1) ^I^ | | 32 (1) ^I^ | | 16 (1) ^I^ | | >512 | | 128 (1) ^I^ | | 64 (0.5) ^S^ | | 64 (1) ^I^ | | 4 (0.5) ^S^ |

AMP: Ampicillin. FEP: cefepime. CHL: chloramphenicol ; KAN: Kanamycin. NOR: norfloxacin. STR: Streptomycin. TET: tetracycline ; CIP: ciprofloxacin. CLX: cloxacillin. ERY: erythromycin. S: synergy. I : indifférence. **( )**: Numbers in bracket are FIC Values

**S2. Fractional inhibitory Concentrations of the association between antibiotics and extracts of *Aframomum citratum* at MIC/2.5 and MIC/5 (µg/ml) against MDR bacteria.**

| **Bacterial strains** | **Antibiotic and FIC** | | | | | | | | | | | | | |
| --- | --- | --- | --- | --- | --- | --- | --- | --- | --- | --- | --- | --- | --- | --- |
|  | **Ampicillin** | | | **Cefepime** | |  | **Chloramphenicol** | |  | **Ciprofloxacin** | |  | **Cloxacillin** | |
|  | **MIC/2.5** | **MIC/5** | | **MIC/2.5** | **MIC/5** |  | **MIC/2.5** | **MIC/5** |  | **MIC/2.5** | **MIC/5** | | **MIC/2.5** | **MIC/5** |
| **AG100Atet** | - | - |  | - | - |  | 0.125 | 0.25 |  | 0.5 | 1 |  | - | - |
| **AG102** | - | - |  | 1 | 1 |  | 0.5 | 0.5 |  | - | - |  | - | - |
| **CM64** | - | - |  | - | - |  | nt | nt |  | nt | nt |  | - | - |
| **KP63** | - | - |  | 0.125 | 1 |  | - | - |  | 1 | - |  | - | - |
| **PA124** | 0.125 | 0.5 |  | 1 | 2 |  | 0.5 | 0.5 |  | 0.25 | 1 |  | - | - |
|  | | | | | | | | | | | | | | |
| **Bacterial strains** | **Erythromycin** | | | **Kanamycin** | |  | **Norfloxacin** | |  | **Streptomycin** | |  | **Tetracycline** | |
|  | **MIC/2.5** | **MIC/5** |  | **MIC/2.5** | **MIC/5** |  | **MIC/2.5** | **MIC/5** |  | **MIC/2.5** | **MIC/5** |  | **MIC/2.5** | **MIC/5** |
| **AG100Atet** | 0.25 | 0.5 |  | <0.125 | - |  | 0.125 | 1 |  | - | - |  | - | 1 |
| **AG102** | 0.5 | 0.5 |  | - | - |  | - | - |  | - | - |  | - | - |
| **CM64** | - | - |  | <0.5 | <0.5 |  | <0.5 | 1 |  | 0.125 | 0.25 |  | nt | nt |
| **KP63** | <0.063 | 0.25 |  | 0.5 | 1 |  | - | - |  | - | - |  | - | - |
| **PA124** | 0.5 | 0.5 |  | 0.125 | 0.5 |  | 0.125 | 0.5 |  | nt | nt |  | 0.25 | 0.25 |

(**-**): Non determined Values of FIC; nt: not tested. There is synergy when FIC ≤ 0.5. indifference when 0.5<FIC < 4. and antagonism. when FIC>4.

**S3. Fractional inhibitory Concentrations of the association between antibiotics and extracts of *Cinnamomum zeylanicum* at MIC/2.5 and MIC/5 (µg/ml) against MDR bacteria.**

| **Bacterial strains** | **Antibiotic and FIC** | | | | | | | | | | | | | |
| --- | --- | --- | --- | --- | --- | --- | --- | --- | --- | --- | --- | --- | --- | --- |
|  | **Ampicillin** | |  | **Cefepim** | |  | **Chloramphenicol** | |  | **Ciprofloxacin** | |  | **Cloxacillin** | |
|  | **MIC/2.5** | **MIC/5** |  | **MIC/2.5** | **MIC/5** |  | **MIC/2.5** | **MIC/5** |  | **MIC/2.5** | **MIC/5** |  | **MIC/2.5** | **MIC/5** |
| **AG100Atet** | - | - |  | - | - |  | 0.063 | 0.125 |  | 0.25 | 0.5 |  | - | - |
| **AG102** | - | - |  | 2 | 2 |  | 0.5 | 1 |  | - | - |  | - | - |
| **CM64** | - | - |  | - | - |  | nt | nt |  | nt | nt |  | - | - |
| **KP63** | - | - |  | 0.125 | 1 |  | <0.063 | - |  | - | - |  | - | - |
| **PA124** | 0.125 | 0.5 |  | 1 | 1 |  | 0.063 | 0.25 |  | 0.5 | 1 |  | - | - |
|  | | | | | | | | | | | | | | |
| **Bacterial strains** | **Erythromycin** | |  | **Kanamycin** | |  | **Norfloxacin** | |  | **Streptomycin** | |  | **Tetracyclin** | |
|  | **MIC/2.5** | **MIC/5** |  | **MIC/2.5** | **MIC/5** |  | **MIC/2.5** | **MIC/5** |  | **MIC/2.5** | **MIC/5** |  | **MIC/2.5** | **MIC/5** |
| **AG100Atet** | 0.25 | 0.5 |  | <0.031 | <0.25 |  | 1 | 1 |  | - | - |  | 1 | 1 |
| **AG102** | 0.5 | 0.5 |  | - | - |  | - | - |  | - | - |  | - | - |
| **CM64** | - | - |  | <0.5 | <0.5 |  | <0.5 | 1 |  | 0.125 | 0.25 |  | nt | nt |
| **KP63** | 0.063 | 0.25 |  | 1 | 1 |  | - | - |  | - | - |  | - | - |
| **PA124** | 0.125 | 0.25 |  | 0.063 | 0.25 |  | 0.5 | 1 |  | nt | nt |  | 0.25 | 0.25 |

(**-**): Non determined Values of FIC; nt: not tested. There is synergy when FIC ≤ 0.5. indifference when 0.5<FIC < 4. and antagonism. when FIC>4.

**S4. Fractional inhibitory Concentrations of the association between antibiotics and extracts of *Dorstenia psilurus* at MIC/2.5 and MIC/5 (µg/ml) against MDR bacteria.**

| **Bacterial strains** | **Antibiotics and FIC** | | | | | | | | | | | | | |
| --- | --- | --- | --- | --- | --- | --- | --- | --- | --- | --- | --- | --- | --- | --- |
|  | **Ampicillin** | |  | **Cefepime** | |  | **Chloramphenicol** | |  | **Ciprofloxacin** | |  | **Cloxacillin** | |
|  | **MIC/2.5** | **MIC/5** |  | **MIC/2.5** | **MIC/5** |  | **MIC/2.5** | **MIC/5** |  | **MIC/2.5** | **MIC/5** |  | **MIC/2.5** | **MIC/5** |
| **AG100Atet** | - | - |  | - | - |  | 0.5 | 1 |  | 0.25 | 0.5 |  | - | - |
| **AG102** | - | - |  | 2 | 2 |  | 0.25 | 0.25 |  | - | - |  | - | - |
| **CM64** | - | - |  | - | - |  | nt | nt |  | nt | nt |  | - | - |
| **KP63** | - | - |  | <0.016 | 0.5 |  | - | - |  | 1 | 1 |  | - | - |
| **PA124** | 0.5 | 0.5 |  | 1 | 1 |  | 0.5 | 1 |  | 1 | 1 |  | - | - |
|  | | | | | | | | | | | | | | |
| **Bacterial strains** | **Erythromycin** | |  | **Kanamycin** | |  | **Norfloxacin** | |  | **Streptomycin** | |  | **Tetracycline** | |
|  | **MIC/2.5** | **MIC/5** |  | **MIC/2.5** | **MIC/5** |  | **MIC/2.5** | **MIC/5** |  | **MIC/2.5** | **MIC/5** |  | **MIC/2.5** | **MIC/5** |
| **AG100Atet** | 0.5 | 0.5 |  | - | - |  | 1 | 2 |  | - | - |  | 1 | 1 |
| **AG102** | 2 | 2 |  | - | - |  | - | - |  | - | - |  | - | - |
| **CM64** | - | - |  | 1 | 2 |  | - | - |  | 0.25 | 1 |  | nt | nt |
| **KP63** | 0.063 | 0.5 |  | 1 | 2 |  | - | - |  | - | - |  | - | - |
| **PA124** | 0.5 | 1 |  | 0.031 | 0.25 |  | 0.5 | 1 |  | nt | nt |  | 0.25 | 1 |

(**-**): Non determined Values of FIC; nt: not tested. There is synergy when FIC ≤ 0.5. indifference when 0.5<FIC < 4. and antagonism. when FIC>4.

**S5.** Fractional inhibitory Concentrations of the association between antibiotics and extracts of *Tetrapleura tetraptera* at MIC/2.5 and MIC/5 (µg/ml) against MDR bacteria.

| **Bacterial strains** | **Antibiotic and FIC** | | | | | | | | | | | | | |
| --- | --- | --- | --- | --- | --- | --- | --- | --- | --- | --- | --- | --- | --- | --- |
|  | **Ampicillin** | |  | **Cefepime** | |  | **Chloramphenicol** | |  | **Ciprofloxacin** | |  | **Cloxacillin** | |
|  | **MIC/2.5** | **MIC/5** |  | **MIC/2.5** | **MIC/5** |  | **MIC/2.5** | **MIC/5** |  | **MIC/2.5** | **MIC/5** |  | **MIC/2.5** | **MIC/5** |
| **AG100Atet** | - | - |  | - | - |  | 1 | - |  | 0.5 | 0.5 |  | - | - |
| **AG102** | - | - |  | 2 | 2 |  | 0.5 | 0.5 |  | - | - |  | - | - |
| **CM64** | - | - |  | - | - |  | nt | nt |  | nt | nt |  | - | - |
| **KP63** | - | - |  | 1 | - |  | - | - |  | 1 | - |  | - | - |
| **PA124** | 0.5 | 1 |  | 1 | 1 |  | 0.125 | 0.25 |  | 1 | 1 |  | - | - |
|  | | | | | | | | | | | | | | |
| **Bacterial strains** | **Erythromycin** | |  | **Kanamycin** | |  | **Norfloxacin** | |  | **Streptomycin** | |  | **Tetracycline** | |
|  | **MIC/2.5** | **MIC/5** |  | **MIC/2.5** | **MIC/5** |  | **MIC/2.5** | **MIC/5** |  | **MIC/2.5** | **MIC/5** |  | **MIC/2.5** | **MIC/5** |
| **AG100Atet** | 1 | 1 |  | - | - |  | 1 | 2 |  | - | - |  | 1 | 1 |
| **AG102** | 2 | 2 |  | - | - |  | - | - |  | - | - |  | - | - |
| **CM64** | - | - |  | 1 | 2 |  | 1 | 2 |  | 0.5 | 1 |  | nt | nt |
| **KP63** | <0.063 | 0.5 |  | 1 | 2 |  | - | - |  | - | - |  | - | - |
| **PA124** | 0.5 | 0.5 |  | 0.5 | 0.5 |  | 0.5 | 1 |  | nt | nt |  | 0.25 | 0.25 |

(**-**): Non determined Values of FIC; nt: not tested. There is synergy when FIC ≤ 0.5. indifference when 0.5<FIC < 4. and antagonism. when FIC>4.
